# Collaborative Computational Project for Electron cryo-Microscopy

**DOI:** 10.1107/S1399004714018070

**Published:** 2015-01-01

**Authors:** Chris Wood, Tom Burnley, Ardan Patwardhan, Sjors Scheres, Maya Topf, Alan Roseman, Martyn Winn

**Affiliations:** a Scientific Computing Department, Science and Technology Facilities Council, Research Complex at Harwell, Didcot OX11 0FA, England; b European Bioinformatics Institute, European Molecular Biology Laboratory, Wellcome Trust Genome Campus, Hinxton, Cambridge CB10 1SD, England; c MRC Laboratory of Molecular Biology, Francis Crick Avenue, Cambridge Biomedical Campus, Cambridge CB2 0QH, England; d Biological Sciences at Birkbeck, University of London, Malet Street, London WC1E 7HX, England; e Faculty of Life Sciences, University of Manchester, Oxford Road, Manchester M13 9PT, England; f Scientific Computing Department, Science and Technology Facilities Council, Daresbury Laboratory, Warrington WA4 4AD, England

**Keywords:** CCP-EM, Collaborative Computational Project for Electron cryo-Microscopy

## Abstract

The Collaborative Computational Project for Electron cryo-Microscopy (CCP-EM) has recently been established. The aims of the project are threefold: to build a coherent cryoEM community which will provide support for individual scientists and will act as a focal point for liaising with other communities, to support practising scientists in their use of cryoEM software and finally to support software developers in producing and disseminating robust and user-friendly programs. The project is closely modelled on CCP4 for macromolecular crystallo­graphy, and areas of common interest such as model fitting, underlying software libraries and tools for building program packages are being exploited. Nevertheless, cryoEM includes a number of techniques covering a large range of resolutions and a distinct project is required. In this article, progress so far is reported and future plans are discussed.

## Abstract 2

The Collaborative Computational Project for Electron cryo-Microscopy (CCP-EM) is a new initiative for the structural biology community, following the success of CCP4 for macromolecular crystallography. Progress in supporting the users and developers of cryoEM software is reported.

## 1. Introduction   

Collaborative Computational Projects (CCPs) are a well established model in the UK for providing long-term support to scientific areas that require significant computation. CCP4 for macromolecular crystallography (Winn *et al.*, 2011 ▶) is of course very well known in the structural biology community. However, there have been over 20 CCPs from CCP1 for quantum chemistry through to the latest CCP-SAS for small-angle scattering (see http://www.ccp.ac.uk). CCPs are characterized by a strong community spirit which encourages pooling of effort in training and in software development.

Electron cryo-microscropy (cryoEM) is having an increasing impact in structural biology. The number of structures being deposited in the Electron Microscopy Data Bank (EMDB) is accelerating rapidly (Lawson *et al.*, 2011 ▶). In fact, cryoEM covers a range of specific techniques, depending on the nature of the sample and the method of collecting images (Saibil *et al.*, 2015 ▶). Improvements in microscopes, detectors and software mean that in favourable cases single-particle cryoEM can provide structural information comparable to crystallography without the need to grow crystals (Li *et al.*, 2013 ▶; Amunts *et al.*, 2014 ▶; Allegretti *et al.*, 2014 ▶). At the other extreme, correlative light electron microscopy (CLEM) provides a link between structural data and cell biology. In all cases, the steps for producing three-dimensional data (molecular volume or tomogram) from two-dimensional images (individual micrographs or tilt series) are computationally intensive.

**Figure 1:**
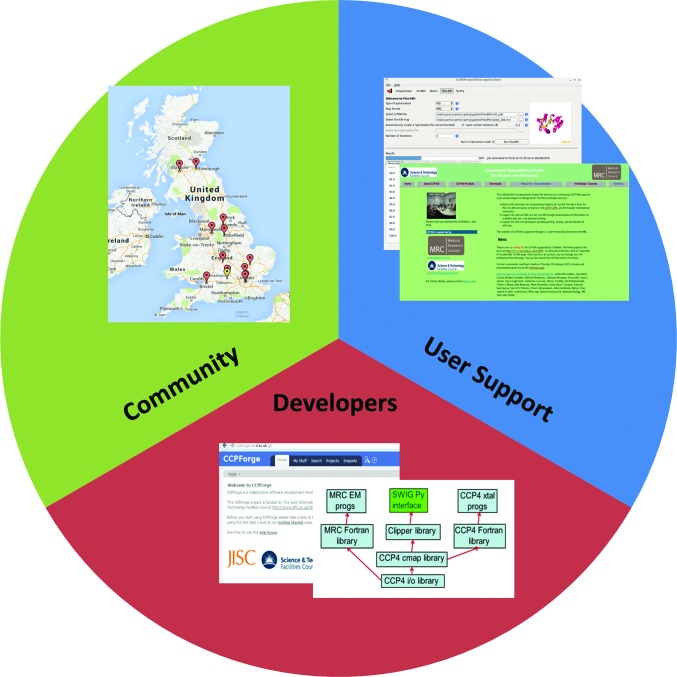
Overview of CCP-EM activities. The green sector refers to community-building activities, and is represented by a map of current groups performing high-resolution cryoEM. The blue sector refers to user-support activities, which are illustrated by the CCP-EM GUI currently under development and a snapshot of the CCP-EM website. Finally, the red sector refers to support of software developers, and is illustrated by the front page of CCPForge, which hosts the CCP-EM software project, and a schematic of the developing CCP-EM library.

Given the computational nature of cryoEM analysis, and the increasing scientific impact, the field was considered appropriate (if not overdue) for the formation of a CCP. An MRC-funded Partnership Grant was obtained with the aim of establishing CCP-EM for biological cryoEM and began in August 2012 (Fig. 1 ▶). The project is coordinated by a core team in the Research Complex at Harwell who have close links to the corresponding CCP4 team. The partnership also includes links to CCPN for biological NMR spectroscopy (Vranken *et al.*, 2005 ▶). In the present article, we describe progress to date and outline our ambitions for the future.

## 2. Building a community for cryoEM   

CCPs are fundamentally intended to be community projects. The longevity of CCP4 is owing in no small part to the succession of chairs, committee members and software developers coming from different laboratories in the UK. It is important that scientists do not see a CCP as ‘somebody else’s project’.

In the case of CCP-EM, we have consulted widely in the UK community and the idea of a CCP is almost unanimously welcomed. Nevertheless, it is still early days, and it would be premature to claim that the community is established. The main CCP-EM-sponsored event so far has been a one-day discussion workshop held in Leeds in February 2013. Attendees from most of the cryoEM laboratories, as well as interested parties from the fields of crystallography and NMR, discussed plans for CCP-EM. Outputs of the day are available on the CCP-EM website (http://www.ccpem.ac.uk), although the most significant output was acknowledged to be the event itself, bringing the different laboratories together in one room.

The purpose of a community is not only self-help, but also to provide a focal point for international discussions. In the case of CCP-EM, this can be illustrated by two examples. The Protein Data Bank in Europe (PDBe) is developing a range of EMDB services for the user community and requires feedback from microscopists to improve these services. At least for the UK, CCP-EM provides a useful user community, and the CCP-EM mailing list has been used to gather feedback on EMDB search filters and a beta-test Fourier Shell Correlation server. At a more technical level, an international grouping of software developers are working on standardizing a set of extensions to the widely used ‘MRC’ file format for image/volume data. CCP-EM, through its connections to the MRC library developers (see below) and to CCP4, which shares the format for electron-density data, is involved in these discussions and will help to disseminate agreed changes to the community.

## 3. Support for users   

CCP-EM will support users partly through the provision of focused training and partly through online resources. As mentioned already, cryoEM actually covers a number of distinct techniques, and in principle all are within the scope of CCP-EM. To be pragmatic, we are focusing initially on single-particle reconstruction, since this is the technique that can benefit most immediately from our links to CCP4. However, we do recognize that electron tomography is becoming of comparable importance and should be included in CCP-EM activities. We also note the existence of a large community of university groups that do not currently have a cryo capability but work with stained samples and face similar computational issues.

The first CCP-EM training event will cover the maximum-likelihood single-particle reconstruction program *RELION* being developed at the MRC-LMB (Scheres, 2012 ▶). The program uses an empirical Bayesian approach with a full statistical model to tune parameters for image alignment and volume reconstruction. CCP-EM now supports *RELION* users *via* the CCP-EM mailing list. Future schools will cover other CCP-EM-associated software, but in the meantime we are planning a workshop on the general computational issues associated with setting up and running a cryoEM laboratory. As for scientists in other areas, one of the major activities is installing a range of software and maintaining in-house scripts which use a mix of programs. Solutions include hiring dedicated support, or outsourcing to third parties (*e.g.* SBGrid), but this is not feasible for all. A related issue is data management, particularly as the size of image sets increases, driven for example by the use of video mode with the newest generation of direct electron detectors (Bai *et al.*, 2013 ▶; Li *et al.*, 2013 ▶). CCP-EM aims to facilitate the sharing of experiences, and the workshop will contribute to this.

In the longer term, CCP-EM will support working scientists though the provision of a user-friendly software suite. The suite is currently being built upon a set of existing programs (see below), which are being integrated into a common environment. The common environment consists of an underlying software library, a Python layer for easy integration and a graphical user interface based on PyQt. These elements form a loose framework that allows the inclusion of a variety of different programs from different authors (following the successful CCP4 model). A common build system is being developed, which will support testing of the suite and the construction of downloadable packages.

A particular strength of CCP-EM is likely to be its close association with the CCP4 project for macromolecular crystallography. ‘High’-resolution cryoEM reconstructions are now of comparable quality to ‘low’-resolution electron-density maps from crystallography, and there is the potential to share many software tools. There are indeed subtle differences, for example X-ray *versus* electron form factors or the distribution of density values over a map, but recent structures clearly show the shape of things to come (Amunts *et al.*, 2014 ▶).

## 4. Support for developers   

As in many scientific fields, software for cryoEM is often written by postgraduate or postdoctoral level scientists in response to an immediate need. There are of course exceptions to this, and some impressive software packages have been built up, for example *EMAN*2 (Tang *et al.*, 2007 ▶); *Spider* (Shaikh *et al.*, 2008 ▶); *Imagic* (van Heel *et al.*, 1996 ▶) and *XMIPP* (Scheres *et al.*, 2008 ▶). Nevertheless, in many cases CCP-EM can add value to community developers by providing long-term support and improved software engineering, and by helping to disseminate software to a larger user community.

*FindEM* is a program for locating particles in an electron micrograph given one or more particle templates (Roseman, 2004 ▶). A correlation map is calculated between the template(s) and the image, and predicted particle positions are displayed in a Tcl/Tk image viewer where thresholds can be adjusted interactively. A procedure for generating templates by averaging hand-picked particles is also included. *FindEM* is now distributed from the CCP-EM website, currently as a stand-alone package. Work is under way to improve the ease of use by re-implementing the pipeline of steps in the portable Python scripting language and by re-implementing the image viewer in the more modern and powerful PyQt language.

As discussed above, *RELION* (Scheres, 2012 ▶) is a program for maximum-likelihood single-particle reconstruction. Although *RELION* provides most functionality for reconstruction, there are some steps that still require manual intervention, such as inspection of the set of two-dimensional class averages and update of the STAR-format metadata files following the rejection of certain classes. CCP-EM is now developing tools for performing such housekeeping steps, and more generally for managing the intermediate data produced during a *RELION* run. These tools are being built upon software libraries which are being developed for CCP-EM, and thus will also help to integrate *RELION* into the wider suite.

*Flex-EM* is a program for the flexible fitting of atomic models into cryoEM volumes (Topf *et al.*, 2008 ▶), and is implemented in *MODELLER* (Eswar *et al.*, 2006 ▶) as a Python program calling core routines. CCP-EM have implemented a GUI to ease the use of *Flex-EM* and to allow pipelining with auxiliary programs such as *RIBFIND* (Pandurangan & Topf, 2012 ▶). The Topf group are also developing TEMPy, which is a Python library file for manipulating cryoEM volumes and atomic coordinates. A particular focus is a set of scoring functions for assessing the quality of fitted coordinates (Vasishtan & Topf, 2011 ▶).

A fundamental method for supporting software developers is by providing software libraries that make developing applications easier. The MRC-LMB have provided a comprehensive software library for EM since the early days (Crowther *et al.*, 1996 ▶), together with a large set of programs and utilities (for example *Ximdisp*; Smith, 1999 ▶). As the principal maintainer of this suite retires, CCP-EM will take over long-term maintenance of this software. The MRC-LMB library is partly based on CCP4 libraries for file input/output. CCP-EM will also adopt these libraries, along with the Clipper library for maps and MMDB (Krissinel *et al.*, 2004 ▶) for coordinates. We have built a comprehensive Python API on top of these libraries using SWIG, which can be called from the integration layer mentioned above.

## 5. Outlook   

There is a general perception that the field of cryoEM has matured such that a wider range of structural biologists are interested in applying it in their studies. Nevertheless, it remains challenging both in terms of the experiment and computationally, and validation of the results is becoming increasingly important (Henderson *et al.*, 2012 ▶). CCP-EM aims to address the computational aspects, adapting the lessons learnt in CCP4 as well as catalysing new solutions to problems specific to the field.

The initiative is timely, as the newly established Electron Bio-Imaging Facility is being constructed at the Diamond synchrotron in the UK. This national facility will provide high-end electron microscopes which can be accessed in the same way as a synchrotron beamline. Computational support and the provision of appropriate software pipelines are important, as they are for X-ray beamlines. The CCP-EM core team, located close by in the Research Complex at Harwell, will help to provide this support. The experience gained here can be transferred to the wider CCP-EM effort.

Structural biology is also becoming more interdisciplinary, and CCP-EM will foster links between communities *via* partnerships with organizations such as CCP4, CCPN and CCPBioSim. Advances such as the development of three-dimensional electron crystallography on protein microcrystals (Shi *et al.*, 2013 ▶) mean that the distinction between techniques is anyhow becoming blurred.

The CCP-EM project has just started, and is currently a much smaller effort than CCP4. Nevertheless, there are reasons to hope that it will grow into a comparable effort if some momentum can be achieved. Such projects can provide great benefits to the scientific community, but also depend heavily on buy-in from the community. We therefore request and encourage active participation from the reader!
